# 16506 Recognizing Interdisciplinary Collaborative Research in Promotion and Tenure Processes

**DOI:** 10.1017/cts.2021.681

**Published:** 2021-03-30

**Authors:** Brenda K. Zierler, Nicole Summerside, Jennifer Sprecher, Erin Blakeney, Mia Vogel, Frances Chu, Jonathan D. Posner

**Affiliations:** 1 Department of Biobehavioral Nursing and Health Informatics, Center for Health Sciences Interprofessional Education, Research, and Practice, University of Washington; 2 Department of Biobehavioral Nursing and Health Informatics, Center for Health Sciences Interprofessional Education, University of Washington; 3 Department of Biobehavioral Nursing and Health Informatics, Center for Health Sciences Interprofessional Education University of Washington; 4 Department of Mechanical Engineering, Department of Chemical Engineering, Department of Family Medicine, University of Washington

## Abstract

ABSTRACT IMPACT: Recognizing Interdisciplinary Collaborative Research in Promotion and Tenure Processes OBJECTIVES/GOALS: Academic institutions have traditionally focused on individual achievements for promotion. We present our effort on identifying and measuring attitudes on promotion and tenure (PT) criteria that values and rewards interdisciplinary research (IR). We have developed a toolkit to facilitate the recognition of IR in PT processes. METHODS/STUDY POPULATION: Our group reviewed appointment, promotion and tenure (APT) policies from each of the six Health Science Schools and the College of Engineering at the University of Washington (UW) to assess language of objective criteria and attributes of IR to guide APT committees in the evaluation of interdisciplinary researchers. We surveyed faculty about their attitudes relating to IR within the context of promotion and tenure. Interviews of department chairs and administrators about institutional policies and infrastructure that supports or inhibits IR, and current best practices, were conducted. We have developed toolkits for junior faculty, department chairs, external reviewers, and APT committees to facilitate rewarding IR at promotion. RESULTS/ANTICIPATED RESULTS: Review of APT policies found criteria that recognizes IR for APT in three schools. 118 faculty responded to the survey (44% Professor, 26% Associate, and 37% eligible for APT committees). The majority of faculty reported they were currently conducting IR (95%), considered IR important (98%), and believed the UW faculty code should encourage IR (85%). Although a vast majority considered their units supportive of IR (>80%), less than half (43%) reported that their APT criteria provided examples that included participation in IR. Our survey also found that APT committees were challenged about best practices to reward IR, APT external reviewers struggle to evaluate individual vs team contributions, and individual faculty are challenged to describe contributions for APT within context of an interdisciplinary team. DISCUSSION/SIGNIFICANCE OF FINDINGS: IR is conducted and valued by UW faculty; however, current structures, policies, and APT code do not facilitate IR for promotion and tenure. We have developed a toolkit for promotion-eligible faculty, chairs, external reviewers, and APT committees to facilitate IR. Our goal is to modify UW faculty code and unit APT criteria to recognize and reward IR.

